# Acute Respiratory Infections

**Published:** 1918-10

**Authors:** H. Zinsser


**Acute Respiratory Infections.** By Major H. Zinsser, M. R. C.

The speaker said he had almost nothing to say about mumps, because he had never made the slightest headway in trying to stop it, and he had come to the conclusion that mumps can be controlled only when we have found a more active bacteriological solution. Fortunately, it is very mild and does not cause a great deal of serious loss.

Pneumonia was very serious in the U. S. when encampments first started. Among other things he noticed a curious change in the character of the disease: whereas, at first, a pneumococcus infection was predominant, later the speaker had seen it change to a streptococcus infection.

All these respiratory diseases should be classified for sanitary purposes in a way different from that in which they have been classified up to the present. The speaker believed we should distinguish between those diseases in which the preventive problem is that of avoiding contact, and those diseases against which we encounter a considerable amount of resistance. In the former class, the problem is that of preventing contact; in the second series it becomes primarily a susceptibility problem or hygiene problem.

The speaker said he would classify as lobar pneumonia all pneumonia caused by pneumococci.

In pneumonia, we are not dealing primarily with a contact problem, but primarily with a hygienic, and secondarily with a contact, problem.

He believed the idea of preventing contact originated in Denmark where, some years ago, a physician isolated his pneumonia cases.

Recently the authorities have sought whether two cases in contact with each other presented the same type of pneumococcus. Examination showed that the bacillus was conveyed from person to person; similar cases have been found in members of the same family.

A tent study of pneumonia in a camp showed a considerable number of cases which seemed attractive for the contact theory. On the other hand, the speaker had found that though they frequently came from the same tent, these cases were not very often of the same type of pneumococcus. In the same tent he had sometimes found a type 1 and a type 2, or a type 1 and a type 4, so he could not logically conclude that he was dealing with a purely contact infection.

One of the artillery companies of this camp was sent at a certain time to a shooting rank. This company arrived while the weather was fine but very soon it began to rain and get very cold, and the shelter was insufficient. After a short time there was an outbreak of pneumonia which amounted to a considerable number of cases, at least eight in a period of 3 or 4 days. These cases were not all of the same type; among them were 2 type 1, 1 atypical 2, 1 type 2, 1 streptococcus type, etc. The associates of these men, 25 in number, were examined for mouth organisms and we found 1 type 1, 1 type 2, 2 atypical 2, 1 streptococcus and influenza, 1 streptococcus, 1 type 4, etc. The point this illustrates is that in order to get lobar pneumonia it is necessary to get pneumococcus in the part affected.

The general conditions, hygienic and sanitary, which reduce the resistance of individuals, are important considerations; what happens in an outbreak of pneumonia is largely a matter of sanitation and hygiene and less directly a matter of inoculation from one individual to another.

The men in the company previously mentioned had been rapidly mobilized; they came from the South and were not accustomed to inclement weather; they were rather crowded and the ventilation at night was not good, because they closed the top of the tents and kept themselves as warm as possible; they had to eat cold food, and after a few days every man had developed a cold; men were coughing and spitting in all the tents. In this way a vicious circle was established : as more of them became carriers, more became susceptible.

In consequence, I believe that to prevent pneumonia of the lobar type, one must not only prevent contact but remember that the most important thing is to avoid reduction of that resistance which naturally is very high in all human beings, and that one is dealing with a matter which requires careful hygiene and sanitation.

The most important of all is the question of ventilation and air space. That, of course, is doubly important, both from the general resistance point of view and from that of increase in carriers.

A few other things to be considered are :

1. The Overwork Factor. There was a very distinct improvement in conditions when the drill was not exhaustive. The method of gradually introducing soldiers to army life is to be recommended and is giving good results; it is a most excellent system.

2. Morning Sick Call. One of the measures adopted in this experience was to try to improve morning sick call. This is very apt to become a routine measure. It is at the morning sick call that the minor infections can best be picked out. In this particular epidemic the speaker and his colleagues obtained the services of a senior medical officer to help the younger officers make their diagnosis. There were a great many cases in which careful examination of the men, who were coughing or showed other signs, prevented the spread of disease. It is therefore, one of the most desirable improvements that can be made.

One cannot, of course, expect too much of a battalion medical officer at the front, not because he is not capable but because of conditions; and the speaker believed in all these sanitary measures one should take the point of view of reinforcement and not that of criticism.

I consider that when we are dealing with the streptococcus pneumonia the problem is entirely different. It is the same sort of problem as in measles: susceptibility is probably universal. The normal resistance of a human adult is much less complete against streptococcus infections than against pneumococcus infections, and while the former are not the same thing as measles, we should regard them as a question of pure contact problems.

*Relationship of Measles with Pneumonia.* — In an epidemic of 701 cases of pneumonia, there were 389 that did not have measles and 312 that presented measles. Of the cases without measles 93 died. The death-rate of primary pneumonia was 12 o/o, and the death-rate of cases following measles was 29.8 o/o. The importance of this is self-evident and it has to do in the first place with the prevention of complications of measles.

As far as measles is concerned there is only one point to bring out, and that is *inspection*. When measles breaks out in a unit the only thing that can help to limit it is frequent inspection; the men should be inspected twice daily if possible. This can be simplified by lining them up and looking at their faces, feeling their pulses, looking at their tongues, and asking them how they are feeling. All cases of cough, conjunctivitis, undue tiredness, etc., should be isolated and observed.

*Meningitis.* — Meningitis falls distinctly in the same category as pneumonia; it is a question of susceptibility. Meningitis is not a disease to which men are universally susceptible; it is not like measles or scarlet fever. It is a disease that one man gets and another does not get under equal conditions, for reasons which we do not understand. The reason for susceptibility is a mystery at the present time.

When meningitis occurs in a command, a number of different methods have been adopted. In the U. S., last year, an attempt was made to make a carrier determination on everyone. In one case an entire division was cultured twice. The speaker did not believe this could be done correctly : to culture 20,000 men twice over in the necessary time is physically impossible. His opinion was that that method is not a good one. There is only one thing in its favor : by making cultures, rough as the methods are, one occasionally picks up the proper carrier and gets the most dangerous men out at once.

Another method is that of making a careful epidemiological study of the men who have been in contact with meningitis, and making a careful bacteriological study of their noses and throats. If one makes careful studies on a limited number of cases this method may be of some value.

In troops that are active, when one cannot determine with whom a particular man was associated, the speaker recommended searching for the carrier rate and, if it goes beyond a certain proportion, treating all as carriers.

I am not sure, however, that the carrier has very much to do with the prevalence of meningitis.

At any rate, I think the two best methods are rate determination and careful bacteriological study of the men who have come in contact with an affected individual.

*Diphtheria.* — In the American army the handling of diphtheria has not been as easy as Colonel Beveridge found it in the British army. This is due to transportation on ships, principally. There is a principle which I should like to speak of as redistribution.

Two or three months ago we had a little epidemic of scarlet fever. A depot battalion had been formed of men from every unit of a certain division; only one of these units had had a case which developed into typical scarlet fever when taken to a French hospital. One man with scarlet fever was sent to the battalion depot, and after 6 days an epidemic began to appear. There were 11 cases in 4 or 5 days, but the disease did not spread in the original unit from where the sick man came.

In the American Forces there has been a great deal of trouble through the system of replacements. When men are continually streaming in, new units are constantly formed and we find that it is best not to disturb healthy units more than is absolutely necessary by forming new units out of old.

In the matter of controlling diphtheria we have been doing cultures on regiments. Diphtheria is easy to handle when you get in a compact unit which is by itself. It can then be stopped absolutely by isolating the entire unit and preventing its mingling with other soldiers and with civilians. The men can then be kept under observation, cultures taken, and the disease controlled in a short time. But this can only be done with limited and stationary units.

Important problems are those of divisions who come over here and where the same vicious circle is established as in pneumonia. Some division begins to get diphtheria and sometimes not a single unit is free from it. It is sometimes 3 or 4 months before you can feel that you have mastered the situation.

The only thing to do is to make careful epidemiological studies of each case and to take all possible sanitary precautions, plus one thing of paramount importance, and that is proper method of washing mess kits.

The usual method is to have a tub of warm, soapy water, and as the men get through with their meals they scrape and wash their mess kits in the tub. The result is that the 150th man gets all the saliva of the others, so if any carriers have left diphtheria bacilli, or any other bacilli, some of the men are sure to get the disease. We have therefore been trying to keep the water boiling, or, if that is not possible, we have given two tubs of water (or more) with a disinfectant solution.

Streptococcus pneumonia and bronchitis were extremely serious in the U. S. last winter. They are arriving here and we shall have more of them next winter. From general experience I think the great thing is to improve sanitation; as sanitation improves, bacteriology becomes less necessary. The ideal would be to eliminate it altogether in favour perfect sanitation.

